# upU-Net Approaches for Background Emission Removal in Fluorescence Microscopy

**DOI:** 10.3390/jimaging8050142

**Published:** 2022-05-20

**Authors:** Alessandro Benfenati

**Affiliations:** 1 Environmental and Science Policy Department, Università degli Studi di Milano, Via Celoria 2, 20133 Milan, Italy; alessandro.benfenati@unimi.it; 2 Gruppo Nazionale Calcolo Scientifico, Istituto Nazionale di Alta Matematica, P.le Aldo Moro 5, 00185 Rome, Italy

**Keywords:** neural network, Perlin noise, U-Nets, particle estimation, deep learning, microscopy imaging

## Abstract

The physical process underlying microscopy imaging suffers from several issues: some of them include the blurring effect due to the Point Spread Function, the presence of Gaussian or Poisson noise, or even a mixture of these two types of perturbation. Among them, auto–fluorescence presents other artifacts in the registered image, and such fluorescence may be an important obstacle in correctly recognizing objects and organisms in the image. For example, particle tracking may suffer from the presence of this kind of perturbation. The objective of this work is to employ Deep Learning techniques, in the form of U-Nets like architectures, for background emission removal. Such fluorescence is modeled by Perlin noise, which reveals to be a suitable candidate for simulating such a phenomenon. The proposed architecture succeeds in removing the fluorescence, and at the same time, it acts as a denoiser for both Gaussian and Poisson noise. The performance of this approach is furthermore assessed on actual microscopy images and by employing the restored images for particle recognition.

## 1. Introduction

The mathematical model underlying the physical process of registering fluorescence microscopy images relies on a linear model

(1) $$\mathrm{gn}=N\left({Hx}^{★}+b\right)$$

where $x^{★}$ represents the clean image, **H** is the blurring linear operator which represents the Point Spread Function (PSF) that introduces perturbation in the recorded image, such as unsharp edges or motion artifacts. The scalar quantity *b* is constant background emission, and gn represents the registered image by the microscope. Equation (1) refers to the discretization of a Fredholm integral of the first kind: the interested reader may find all the technical details in the seminal work [1]. In Equation (1) N(·) represents the noise affecting the image: in case of additive noise Equation (1) reads as

(2) $$\mathrm{gn}={Hx}^{★}+b+\eta$$

where η is a Gaussian variable of zero mean and variance equal to $\sigma^{2}$. In this case the noise is statistically independent of the data. A further noise affecting microscopy images is Poisson noise, which models the errors done in counting processes: indeed, the image acquisition process consists in counting the photons hitting the Charge-Coupled Device (CCD) of the camera. Such kind of noise is signal–dependent: low values on ${Hx}^{★}+b$ induce a high level of noise, while Poisson noise is not highly disruptive when the values of the recorded image are large. Typical microscopy images are affected by both noises: the Gaussian statistics models the so–called *read noise*, which is the perturbation due to the electrical components of the camera, whilst the Poisson noise (called also *photon noise*) models the error on the photon–counting process. The  details of the physical process and the subsequent mathematical treatment can be found in [1,2].

A classical approach for the restoration of microscopy images described by Equation (2) relies on a variational framework [1]: an estimation of $x^{★}$ is obtained by solving an optimization problem involving the sum of two functionals. The first term is the *fit–to–data* or *data fidelity*, which measures the discrepancy between the registered data and the recovered image. The choice for this functionality depends on the noise affecting the data: in presence of Gaussian noise the most suitable one is the Least Square functional [3], whilst in presence of Poisson noise the (generalized) KullBack–Leibler function [4,5] is widely employed. The second term occurring in the minimization is the so-called regularization term, which has the role of considering some characteristics of the desired image and controlling the influence of the noise in the reconstruction. Several options are available for such function: $\ell_{2}$ norm for diffuse components [6], $\ell_{1}$ for promoting sparse solution [7,8], Total Variation functional [9,10,11] (or its smooth counterpart [12]) for edge preserving. One may consider a composite regularization function, such as the Elastic–Net [13], or even non-convex options are available [3,14]. The sum of the data fidelity and the regularization function is balanced by the so-called regularization term, whose estimation is still an open problem [15,16]. Some mathematical manipulation of the regularization function [17,18] or the entire functional [19] may lead to remarkable results. Modern techniques aim to couple classical variational approaches with Deep Learning techniques [20,21,22].

In some cases the term *b* is not constant, and it actually models various degrading contributions: undesired emission from auto–fluorescent objects (i.e., molecules, beads), fluorophores that spontaneously emit light but that are not the actual target of interest, the reflection of the light used to investigate the sample ([23], Figure 2), diffraction due to the medium surrounding the objects of interest. Merging all the above observations on the influence of the statistical noise on the recorded image, one can hence write then Equation (2) as

(3) $$\mathrm{gn}=P\left({Hx}^{★}+b\right)+\eta$$

where P(·) models the Poisson noise, η is a Gaussian random variable of zero mean and variance $\sigma^{2}$ and **b** is no more a scalar but an array of suitable dimension that models the background emission. In some applications, such as particle position estimation [24,25,26], the presence of this artifact may worsen the performance of the algorithms employed for this task. Moreover, in order to test such algorithms, one must have at their disposal a large number of microscopy images with ground truth data to properly assess the performance of the developed strategy. One way to achieve this is to generate synthetic data: the blurring operator **H** can be modeled by a Gaussian PSF or by an Airy Disk [27,28,29], while the statistical noise (both Gaussian and Poisson types [30]) may be simulated by established software procedures.

The auto-fluorescence noise can be simulated in various ways. In [31] the authors employ a combination of vector and scalar-based PSF, polarization detection strategy, spectral crosstalk, and other physical phenomena. Montecarlo methods have been widely used in the literature [32,33,34], both for the reliable approximation of the light scattering and for the simple numerical implementation. The authors in [35] develop a quasi–analytical model for improving the brute force approach based on Monte Carlo methods. Intermittent fluorophores activation was considered in [36], where the spatial distribution of such fluorophores is sparse. The author of [37] analyses the theoretical framework of emission and scattering phenomenon in fluorescence microscopy.

**Figure 1:**
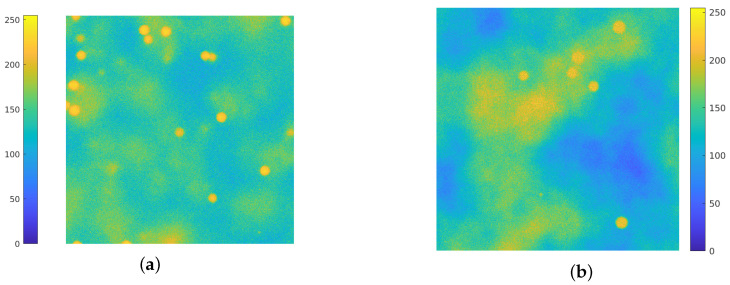
(**a**): an actual fluorescence microscopy image, of dimension 512×512. (**b**): a synthetic image, where the background fluorescence is simulated via Perlin noise. Both images are displayed with values in [0,255].

In this work, the simulation of the background emission is based on the employment of Perlin noise [38]. This procedure is widely used on CGI (Computer–Generated Imagery), it has been employed to simulate clouds in satellite images [39,40,41] and recently has found several applications for the generation of synthetic microscopy images. In [42,43] Perlin noise at different frequencies is added to the ground truth images for the simulation of the background straining. The authors in [44] use a combination of Perlin noise and Gaussian functions for inserting diffuse spots of fluorescence in the background of synthetic images. Due to its flexibility and ability to simulate realistic texture in biological images, Perlin noise is employed for simulating both background emission and cell textures [45,46,47,48], and in [49] a combination of Voronoi diagrams and Perlin noise is used to generate realistic cytoplasm boundaries. In Figure 1, a comparison between an actual image (Figure 1a) and a synthetic one is provided (Figure 1b): the Perlin noise indeed provides a concrete model for the background emission. Recent works [50,51] develop a BGnet architecture to efficiently remove the structured background: the authors employ the Perlin noise for two main reasons: firstly, because it resembles the structured background that can be encountered under real–world experimental conditions and, secondly, because its structure can be controlled by properly setting its spatial frequency among the octaves.

To the best of the author’s knowledge, there is no developed variational approach for background emission removal: in this work, a completely data–driven procedure is developed for the background emission removal. The proposed strategy relies on Deep Learning techniques, namely on U-net architectures.

U-net architectures consist of two main structures: the first one has a contracting behavior, and it aims to analyze the input data at different levels. It corresponds to the encoder (or analysis path) of traditional autoencoders (AEs), and in the original work [52] this path was developed for searching for meaningful information for segmentation task. It presents several levels, and each level lowers the dimension of the input: for example, a contracting path with 5 layers may reduce a 256×256 image to a 128×128 at the first level, then to 64×64 at the second and the last later will output an 8×8 image. Usually, each stop of the contracting path halves the dimension of the input.

The second part of a U-net consists of the expansion path, or synthesis path, which corresponds to the decoder of an AE. The original aim of this part is to learn localized information for pixel–classification. The layers of this path will increase the size of the output, reaching at its final layer the original dimension. Moreover, each level of the contracting path is connected with the relative layer in the expansion path: this allows us to propagate information through the network. The symmetric u–shape gives the name to this type of network. The interested reader may find complete reviews on U-Nets and their application in medical imaging in [53,54].

Such architectures have been introduced to biomedical imaging in the seminal work [52] and revealed to possess remarkable performances in segmentation tasks [55,56,57] but also in generating synthetic images when coupled with Generative Adversarial Networks (GANs) [58]. To the best of the author’s knowledge, the approach of applying U-net like architectures for removing background emission in microscopy imaging is novel: the main idea is to provide as an input to the neural network a corrupted image, namely gn from Equation (3), and require as output an image cleansed by the background emission, namely $P{(Hx}^{★})+\eta$. Due to the typical behavior of U-net-like architecture and autoencoders, one expects smoothed images as outputs: this is the reason why the neural network is trained with noisy images as output and not with completely clean images.

The rest of the paper is organized as follows. Section 2 depicts the architecture employed for addressing the task of background emission removal and Section 3 provides the details of the synthetic data generation. In Section 4 numerical experiments are carried on to assess the performance of the proposed procedure on images that are blurred by a Gaussian PSF and which are affected by Gaussian, Poisson, and Perlin noise. Moreover, this procedure is tested on actual microscopy images and an application to a practical problem, namely the particle’s position estimation, is shown. Section 5 draws the conclusions and presents the future perspectives of this work. The code used in this work is available on https://github.com/AleBenfe/upU-net_Perlin (accessed on 13 May 2022).

## 2. upU-net Architecture

**Figure 2:**
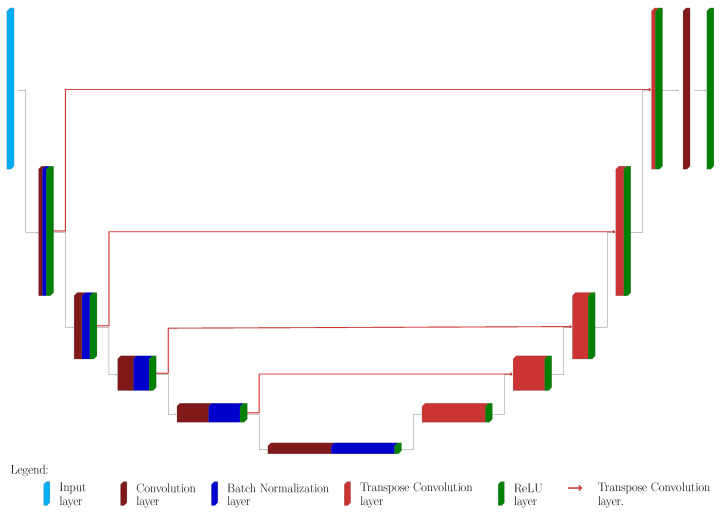
The Proposed upU-net architecture. The contracting path consists in several blocks, 5 in the example provided in the Figure. Each block is composed by convolution layer (dark red), batch normalization layer (dark blue) and a Rectified Linear Unit (ReLU) layer (green). The expansive path’s blocks have just two parts: up-convolution layer (light red) and a ReLu layer. All the features of the last block are merged via a 1d1 convolution layer, followed by a further ReLu. The red lines are connecting the outputs of each block from the contracting path with the block of the expansive path of higher dimension: this seems to induce a smoother behavior of the net.

**Figure 3:**
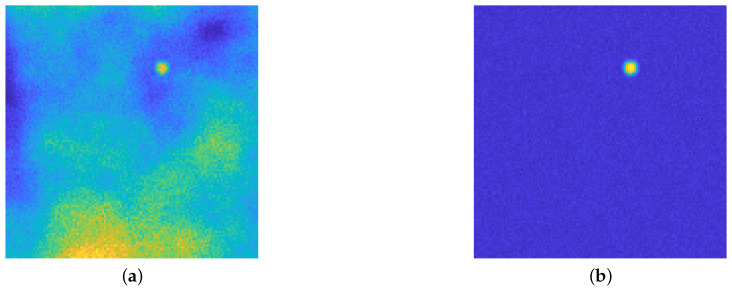
(**a**): the acquired image, affected by several artifact due to the blurring operator, Gaussian and Poisson noise and background emission, modeled by Perlin noise. (**b**): the desired output. Both images are displayed with values in [0,255]. The aim of the proposed upU-net is to remove only the Perlin noise: the experimental results will show that the typical behavior of such AE–like architecture will remove also the statistical noise. See Section 3 for the details about the generation of such image.

The proposed architecture is a slight modification of classical U-Nets and it is depicted in Figure 2. The main difference is that it is not employed for the classification task, but for Perlin noise removal: Figure 3 shows the original image and the desired output image, where the background emission is not present anymore, while the statistical noise is conserved.

The upU-net presents a contracting and expanding path: each block of the former consists of three different layers:

- A convolution 2D layer, with stride 2 (in both dimensions) and padding 1 (in both dimensions). The boundary conditions are set to symmetric, excluding the image edge.
- A batch normalization layer.
- A ReLU layer.

The dimension of each convolution layer is $3\times 3\times 2^{2+l}$, where *l* is the block level: for example, for a net with 5 blocks, the activation of the convolution layer of the last block is then 3×3×128.

The synthesis path of the upU-net architecture presents the symmetric blocks, each one consists in

- An up–convolution layer. The scope of this type of layer is to increase the size of the input, and the last one will provide data of the same size as the input.
- A ReLU layer.

The actual task of up-convolution layers is to increase the size of the input by performing an interpolation: training the neural network means that such interpolation is not *a–priori* selected (e.g., linear or cubic), but the weights and the bias are learned, tailoring the network to the particular data in consideration. ReLU layers are employed to assure non–negative data through the network: this is a physical requirement, since images (RGB or gray–level ones) have non-negative pixel values. The last two layers consist in a 1×1 convolution layer, in order to collapse all the filters in the 2D image, and in a final ReLU layer to ensure again non-negative values on the final output.

The major novelty presented in this approach is that the connections between the corresponding blocks of the two paths do not follow the classical scheme. Indeed, the block at level *l* in the contracting path is connected to the (l+1)-th block of the expansive path, and not to the related *l*-th one, and this connection is made via other up–convolution layers (see Figure 2). In this way, the propagation of information is smoother, and the reconstruction will suffer less from the presence of statistical noise, since the up–convolution layers act as smoothing filters.

**Figure 4:**
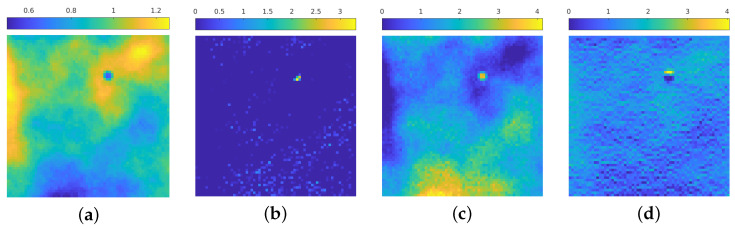
(**a**–**d**): 4 out of 8 activation of the 1st block in the contracting path. The images are displayed in different color scale, since the ranges of the activation values differ by a large amount between filters.

Figure 4 presents a visual inspection of some activations of the net trained in Section 4.1 for each block of the contracting path for the image in Figure 3a: we see that the first blocks aim to discern the areas in which the emission background has more influence, and at the same time can roughly recognize the position of the isolated particles and recognize the presence of statistical noise. In particular, Figure 4a recognizes the areas in the image where the emission background is not present, at the same time Figure 4c shows this activation can find the areas in which such emission is stronger. Figure 4b seems to find the position of the particle, still in a very approximate way; Figure 4d shows that the neural network, at this stage, can find the zones in which the statistical noise is predominant: indeed, the higher activation corresponds to the dark areas in the original image, where Poisson noise is present at a higher level due to low photon count.

**Figure 5:**
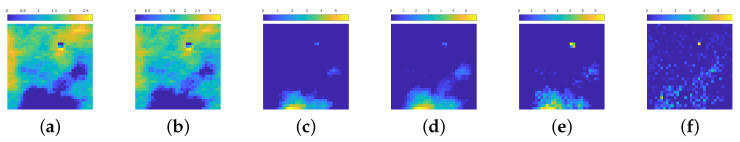
(**a**–**f**): 6 out of 16 activation of the 2nd block in the contracting path. The color scale is not the same among the images, due to different value ranges among the filters.

This behavior is maintained also in deeper blocks, as confirmed by the inspection of some of the activations of the second block in Figure 5. The net tries to separate the zones in which the emission background is present, while searching for the position of particles, if any, and recognizing the presence of statistical noise.

## 3. Data Generation

The synthetic images employed in the numerical test of Section 4 are generated as follows.

**Figure 6:**
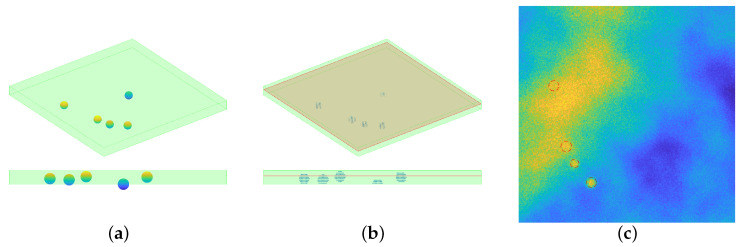
Visual inspection for the synthetic data generation. (**a**): the particles’ position is randomly chosen in a continuous space, and the green shaded volume represents the volume taken into consideration. Notice that a particle may not be completely included in the volume, see the bottom part of the panel (**a**). (**b**): once the resolution is chosen, the particles volume is discretized and only the voxels inside the volume are considered. One slice of the whole volume is considered: such slice is perturbed with Perlin noise, then the action of a PSF induces a blur effect and eventually Gaussian and Poisson noise are added. (**c**): the final simulated image, displayed with values in [0,255]. The dashed red lines represent the profile of the particles.

The position of *p* particles of radius *r* is randomly drawn, from an uniform distribution, in a continuous volume if dimension $D_{x}\times D_{y}\times D_{z}$. The procedure checks that there is no intersection between the particles, given the position and the radius. The volume is then discretized, by choosing the resolution of the final image: denote with $n\times n\times n_{z}$ the number of voxels on each dimension, then each voxel will cover a volume equal to dx×dy×dz, being $dx=D_{x}/n,\;dy=D_{y}/n,\;dz=D_{z}/n_{z},\;$. The horizontal frames are assumed to be square. Each voxel within a distance less than *r* is set to 229.5. This particular value comes from the choice of setting the value of the beads equal to 90% of the maximum value registered: since the aim is to simulate tiff images, such value is 255. One horizontal slice is then considered a registered image: this choice has been made for avoiding to have circular profiles with the same radius. Indeed, since the particles have been randomly positioned in a 3D space all the beads’ centers will have different *z* coordinates: this means that considering just one slice of the 3D volume allows to intersect the particles at different heights, thus in a single frame the profiles will have a radius that ranges from 0 (no intersection at all) to 2 μm (maximum intersection at the equator). The steps for particles’ simulation are depicted in Figure 6.

A Perlin noise [38] pattern is then added to the image: the generation of this perturbation is pursued by following the improved procedure described in [59,60]. Each instance of the noise is generated by using 8 octaves, an initial frequency of 1, an initial amplitude of 1, and persistence of 0.5. At each octave added after the first, the frequency is doubled and the amplitude is multiplied by the persistence.

A Gaussian PSF with variance $\sigma^{2}$ is employed to blur the image, for simulating the physical process of image acquisition in fluorescence confocal microscopy.

The last steps regard the statistical noise perturbation:

- Gaussian noise is added following the procedure depicted in [61]: a multivalued random Gaussian variable η is generated, then a noise level $\sigma_{n}$ is chosen. Being **g** the noise–free image, the perturbed one is then
  (4) $$\mathrm{gn}=g+\sigma_{n}\frac{\eta }{{∥\eta ∥}_{F}}{∥g∥}_{F}$$
  where ${∥\cdot ∥}_{F}$ denotes the Frobenius norm. In this way, one has that ${∥g-\mathrm{gn}∥}_{F}=\sigma_{n}$.
- Poisson noise is added, using built-in functions of the software used (see Section 4 for details).

## 4. Results

This section is devoted to assessing the performance of the proposed approach. All the experiments were done on a Linux machine (Ubuntu 20.04) with an Intel(R) Core(TM) i5–8250U CPU (1.60 GHz), 16 GiB RAM (Intel, Santa Clara, CA, USA) and under MATLAB R2022a environment (MathWorks, Natick, MA, USA), employing the Parallel and the Deep Learning toolboxes [62,63]. The code is available at https://github.com/AleBenfe/upU-net_Perlin (accessed on 13 May 2022). The first run of experiments is devoted to testing the proposed architecture on small-size images and a comparison with a more classic U-net architecture is pursued. The third part of this section is aimed to test the net on a different dataset to the one it was trained on, and Section 4.4 and Section 4.5 are devoted to employing the reconstruction obtained by the network for particle estimation problems. Finally, the last part shows the achieved results on real images.

### 4.1. Assessing the Performance of upU-net

The first experiment is carried out on small–sized images: this has been done for providing the proof of concept of the reliable performances of the proposed method. The architecture employed in this experiment has the following structure:

- 5 blocks in the contracting part, each one containing a 2D convolution layer, a batch normalization, and a ReLU layer (see Figure 2). The number of filters in the first block is 8, then it is doubled in each subsequent block.
- 5 blocks in the expansive path, consisting of an up–convolution layer and a ReLU layer. The number of filters starts from 128 and then it is halved, mimicking the structure of the contracting path.
- 5 up-convolution layers that connect the relative blocks in the two paths.
- A final 1D convolution layer for merging all the filtered data, coupled with the last ReLU layer.

The dataset for this experiment contains 500 images: 90% of them are used for the training, 5% for the validation, and the remaining 5% as the test set. All the images are blurred by a Gaussian PSF with σ=2 and they are affected by Gaussian noise with $\sigma_{n}=0.03$ and by Poisson noise. The training is done by giving an input to the noisy image net (see Figure 3a) and requiring as output Perlin-free images (see Figure 3b). The loss function employed for the training is the least square function. The chosen solver is the ADAM algorithm [64] with default settings, with an initial learning rate of 0.01, a drop factor of 0.2 every 25 epochs with a piecewise constant strategy, a minibatch size of 50 items, and 200 maximum epochs. Moreover, the loss function consists of a regression function and it is augmented with an $\ell_{2}$ regularization term, balanced by a parameter equal to 0.1.

The dataset for this experiment has been generated under the following setting. A volume of 76×76×5μm has been considered, and a random number of particles between 10 and 15 is randomly chosen. The particles’ radius is set to 2μm, and the single horizontal frame has 128×128 pixels. Each slice is perturbed with Perlin noise, then a Gaussian PSF with variance equal to 2 is used to induce a blur effect on the image. Subsequentely, Gaussian noise at level 3% is added to the image and the MATLAB’s function imnoise is employed for adding Poisson noise (a scaling of $10^{-12}$ on the input and a subsequent re-scaling by $10^{12}$ is pursued in order to obtain reliable results: the interested reader should check the function documentation for more technical detail about this scaling [65]).

**Figure 7:**
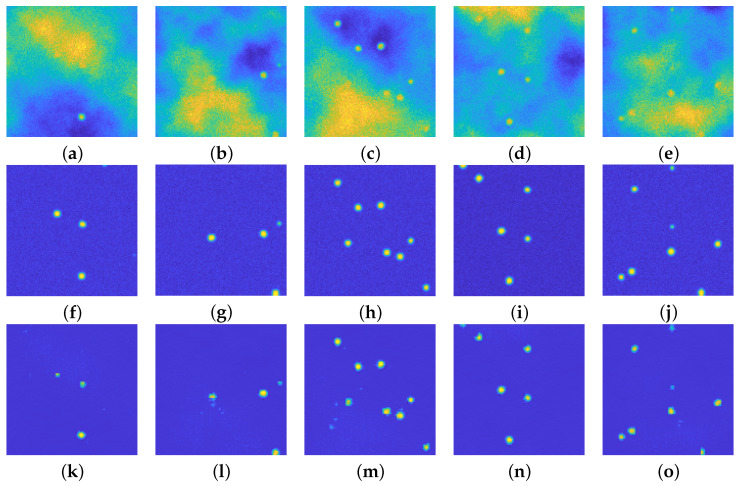
(**a**–**e**): Perturbed images. (**f**–**j**): relative ground truths. (**k**–**o**): recovered images via the proposed approach. In each of the 5 example cases, the particles are well recognized, even the faint ones. Small artifacts arise (see (**l**,**m**) due to the presence of background emission of high intensity. All the images are displayed with values in [0,255].

Figure 7 presents the results of the first run of tests on some items of the test dataset: the first row presents the noisy images, the second row the ground truth and the last one shows the reconstruction obtained by the proposed architecture.

All the reconstructed images present a smoother background with respect to the ground truths: this is not surprising, as one expects exactly this kind of behavior when using an autoencoder–like structure, such as the proposed one. It is quite clear that the background emission is completely removed from all the images. Moreover, the blurring effect is still present in the recovered images, since the upU-net is trained on blurred ones.

The first image (Figure 7a) seems to present just one particle: the ground truth in Figure 7f reveals that there are two more particles in the diffuse, yellow area. The upU-net is capable to recognize the presence not only of the most visible bead, but also of the two hidden particles, even if their shape is not completely circular.

The second image (Figure 7b) presents three main issues: one hidden particle in the diffuse area (as in the previous case), one faint particle on the right boundary of the image, and one bead close to the bottom yellow cloud. As one can see in Figure 7l, the upU-Net scheme can recognize the presence of both the hidden and the faint particle, and at the same time it does not confuse the bead at the bottom with the emission background.

Figure 7c does not present particular problems: indeed all the particles are well recognized, but it is evident that a small artifact may arise, even if it is rare. Figure 7n shows again as upU-net is capable to recognize particles close to intense emission, whilst Figure 7o shows that faint particles merged inside low-intensity background emission can be easily recognized.

### 4.2. Comparison with Classical U-net

This section is devoted to assessing the difference in performance between the proposed approach and a more classical U-net architecture.

A U-net architecture with 3 layers of depth is firstly generated by using the unet MATLAB command: a slight modification of the output of this function is pursued, such modification consists in inserting a batch normalization layer between the ReLU layer and the successive convolution one. This addition is required otherwise the network, in its original form, is not able to learn any parameters and outputs only zeros images. The encoder’s structure consists of 3 blocks, each one containing

- a convolution layer
- a ReLU layer
- a batch normalization layer
- a convolution layer
- a ReLU layer
- a batch normalization layer
- a max-pooling layer

The deepest level of the contracting part presents a dropout layer with a probability of dropout of 0.5, before the pooling. The decoder’s part consists in

- an upconvolution layer
- a ReLU layer
- a concatenation layer, for connecting with the relative encoding part
- a convolution layer
- a ReLU layer
- a batch normalization layer
- a convolution layer
- a ReLU layer
- a batch normalization layer

The bridge structure of the U-net has the same structure as an encoder’s block, i.e., 2× (a convolution layer + ReLU + batch normalization), without the max pooling operation. The initial number of 3×3 filters is 8.

**Figure 8:**
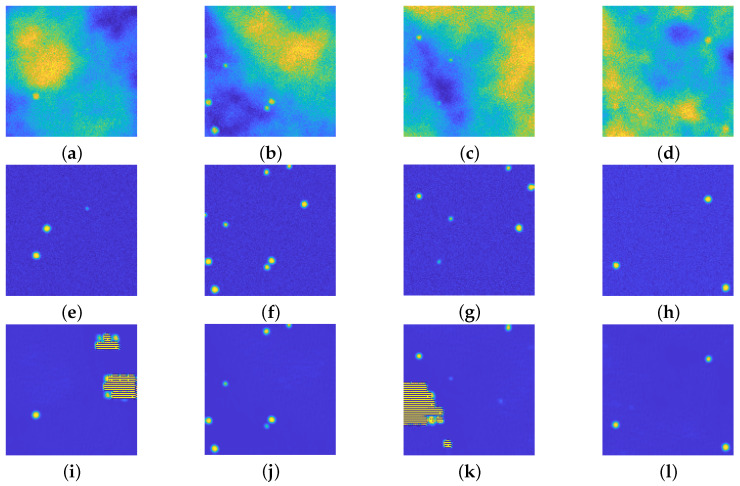
(**a**–**d**): Synthetic images. (**e**–**h**): ground truths. (**i**–**l**): recovered images via the U-net. This network is able to recover some of the images, but unfortunately it introduces some undesired artifacts. All the images are displayed with values in [0,255].

Such network is trained on the 128×128 image dataset, the choice of 3 levels of depth is done to have the same number of learnable parameters, with respect to the upU-net used in Section 4.1: approximately 295,500 the former, approximately 121,300 the latter. The first difference between the proposed approach and the U-net is the training time: the upU-net required around 15 min whilst the U-net required around 55 min, despite having a low number of learnables. In terms of achieved results, the U-net introduces artifacts in some of the recovered images, as evident in Figure 8.

The higher computational time required for training (despite its lower number of parameters to be learned) and the rising of such artifacts in the recovered images show that the proposed upU-net is a valid competitor of classical U-net approaches.

### 4.3. upU-net for Different Radius Particles

In this section, another upU-net is trained for recovering images of size 256×256: the training parameters are the same as the net studied in Section 4.1, whilst the structure of the net consists of 4 blocks instead of 5. The training dataset has the same structure as the one used in Section 4.1, the sole difference is the dimension of the images. The training time was around 40 min.

**Figure 9:**
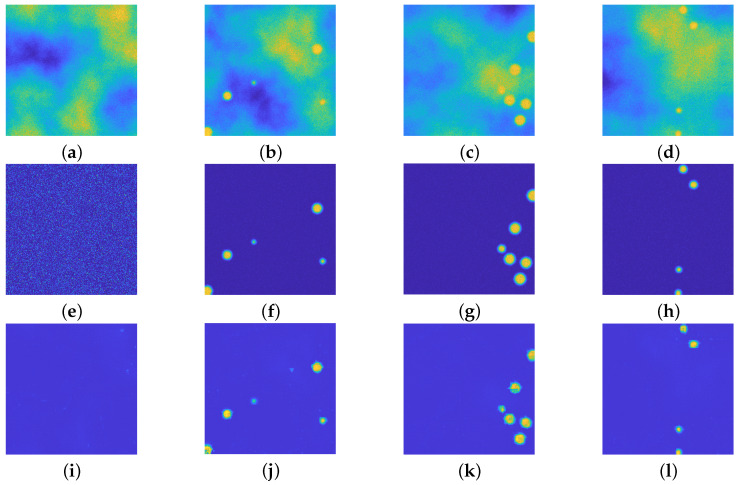
(**a**–**d**): Synthetic images with beads with radius in [0.5,4] μm. (**e**–**h**): ground truths. (**i**–**l**): recovered images. he upU-net is able to reconstruct images with particles with different radius with respect with its training. All the images are displayed with values in [0,255].

Another dataset is then created, where the number of particles ranges from 0 to 15 and where their radius lies in the interval [0.5,4]μm. Figure 9 presents the visual inspection of the achieved results: even if the net has been trained in images generated by volumes with particles of the same radius, it can reconstruct beads of different radii. This is due to the fact that each image of the training dataset is generated by considering a frame intersecting the volume at a fixed height, hence the registered profiles have a different radius (see Figure 6 for a detailed visual inspection).

### 4.4. upU-net for Particle Estimation Task

This last run of experiments refers to the estimation of the particle position when the background emission heavily affects this task.

**Figure 10:**
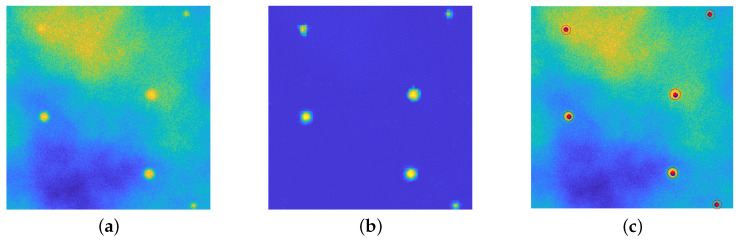
Particle Estimation task. (**a**): original image. (**b**): recovered image by upU-net. (**c**): superposition of the true particles’ center (blue dots), of the estimated center (red dots) and of the estimated profile (red lines). The values range in the interval [0,255]. In this situation one has several particles that are easily recognizable, a faint one (in the top right corner) and a last bead inside a diffuse emission area (top left corner.)

Beside the upU-Nets presented in Section 4.1 and Section 4.3, a further one is trained for recovering 512×512–size images. The training parameters are the same used for the previous nets, the sole change regards the number of epochs, fixed to 60: this leads to a training time of about one hour. Moreover, further 100 synthetic images with dimensions of 128,256,512 are generated, slightly different from the ones used for training and validation. In this new dataset the Perlin noise has a large influence on the final registered data. The task of recognizing the particles’ center is pursued by employing the algorithm depicted in [66], slightly modified and tailored for handling 2D images. Figure 10 shows the potentiality of the proposed procedure: particles embedded in large portions with background emissions with high intensities are easily recognized. The employed procedure, moreover, allow us to estimate both the center and the radius in the frame. Due to some distortion provoked by the reconstruction of the upU-net, the exact position is not completely recovered: anyway, there is a clear reckoning of the presence of particles even in difficult imaging situations.

The procedure depicted in [66] allows also to assess the performance of the upU-net approach. Two measurements are employed for each image: the True Positive Ratio (TPR)

(5) $$\mathrm{TPR}=\frac{\#\{E_{p}\cap P\}}{\#\{P\}}$$

where $E_{p}$ is the set of estimated particles and *P* is the set of actual particles present in the image. TPR measures the fraction of the recognized particles that are actually in the frame in examination. Given a set *A*, #A stands for the number of elements of the set *A*. The second measurement is the number of false particles

(6) $$\varphi =\frac{1}{N}\sum_{i=1}^{N}\#\left\{F_{i}\right\}$$

where $F_{i}$ is the set representing the false particles recognized in the *i*-th frame. The quantity in Equation (6) represents then the average number of recognized particles that are not actually present in the dataset, and *N* is the number of images in the dataset. Table 1 show the performance of the nets.

The average TPR on all datasets (100 images each) is greater than 90%, whilst the average number of particles erroneously recognized is less than one in each test case: this means that the total number of particles is slightly underestimated, and it is very rare that the artifacts are recognized as beads in the image.

### 4.5. Volume Reconstruction

A further experiment considers the reconstruction of the particles’ position in 3D space. A volume of 76×76×6 is discretized in an array of 512×512×22 voxels, and several particles are randomly put in such volume. Each registered frame is processed by the appropriate upU-net, the procedure can be summarized in two steps:

1. Apply to each frame the up–UNet, in order to remove the noise artifacts.
2. Apply the procedure in [66] to the restored array.

**Figure 11:**
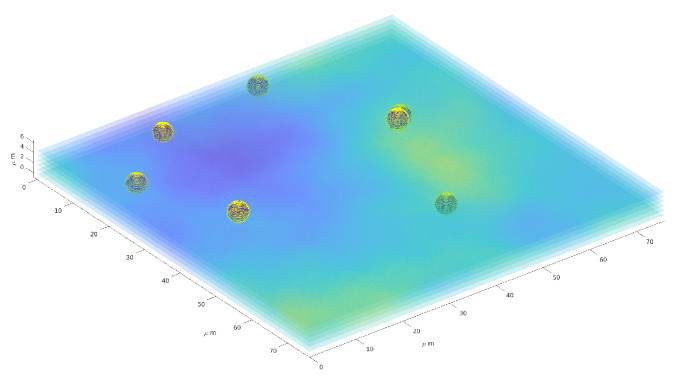
Three–dimension reconstruction of particles distribution. Six layers (out of 22) are superimposed to the sphere representing the particles.

Figure 11 provides a three–dimensional visual inspection of the same procedure applied to one reconstruction of the particle distribution in space, with a superposition of some of the layers of the 3D image.

### 4.6. Real Dataset

**Figure 12:**
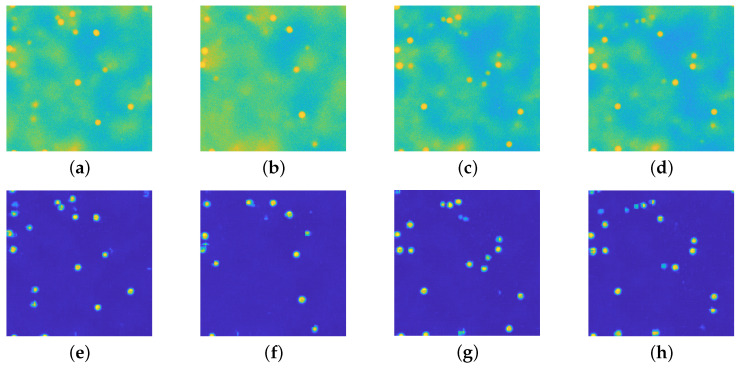
(**a**–**d**): registered images displayed in [0,255]. (**e**–**h**): recovered images via the upU-net architecture displayed in [0,255]. The fluorescence has been successfully removed and the particle position is evident from the restored images.

This section is devoted to presenting the performances of the proposed architecture on real images. The microscopy images refer to a 3D volume with dimension $D_{x}=D_{y}=64\;\mu$m, $D_{z}=4.1\;\mu$m which has been discretized in a 3D array of 512×512×10 voxels. The radius of the beads is 1.5 μm and they are suspended in a ∼70–30% glycerol/water mixture (viscosity of approximately 0.017 Pa s). The microscope used to acquire this data is aZeiss LSM 700 with a 100 × NA 1.4 oil immersion objective (Zeiss Plan–APOCHROMAT) (ZEISS, Oberkochen, Germany). The volume has been scanned 50 times, and the acquisition time for each frame is 0.5 s. A visual inspection of the obtained results is in Figure 12: the background fluorescence emission has been successfully removed, the beads’ profiles are evident in the recovered images. This experiment provides empirical proof that Perlin noise does simulate in a reliable way the fluorescence emission, even if its connection with the actual physical phenomenon is faint.

## 5. Conclusions

This work presents the novel application of a U-net–like architecture for removing background emissions from fluorescence confocal microscopy images. The background emission is simulated by Perlin noise, which has a high degree of similarity with the actual emission. Moreover, the images were perturbed with Gaussian and Poisson noise, after having been blurred via a Gaussian PSF. Such realistic images, with different dimensions, were then used for the training and the test of the upU-net architecture: the visual inspection of the results of both experiments presents a remarkable performance, showing that the proposed approach efficiently removes the background emission and at the same time it exploits the typical behavior of autoencoders, i.e., the recovered images present a more smooth appearance and the both Gaussian and Poisson noise are removed. A comparison with classical U-net approaches shows that the proposed strategy overcomes the classical one, due to its robustness, its performance, and its lower computational time for reaching convergence. The proposed procedure is then coupled with a particle estimation algorithm: the numerical experience showed that the performance of reckoning the beads in a microscopy image is greater than 90% and moreover there is a really low probability that artifacts are mistaken for actual particles. Finally, the procedure is applied to real images. This last experiment provides two main pieces of evidence: Perlin noise is a valid candidate for simulating the background fluorescence noise and that the upU-net can remove such kind of perturbation also on real microscopy images.

This work may pave the way for several new approaches. On one hand, the Perlin noise can be investigated more for the simulation of the fluorescence and the background emission, by encompassing some physical properties in the generation of the noise, such as the presence of the particles. Moreover, instead of considering a simple 2D frame, 3D data can be easily generated, employing the same procedure for the simulation of 3D Perlin noise (see Section 4.5) and convolution techniques for 3D PSFs. A further investigation of the theoretical aspect regarding the reasons why this particular architecture provides such good results will be the object of future work. The neural network architecture can be furthermore employed in more general imaging problems: for example, the encoder part can be employed as the regularization term [67] in a variational framework, or the recovered images ease a segmentation task [68].

## Figures and Tables

**Table 1:** Performance assessment of the proposed procedure in view of particle estimation task. TPR is the true positive ration depicted in (5), while φ is the average number of particles erroneously recognized (see (6)).

Image Size	TPR	φ
128×128	90.87%	0.1
256×256	90.19%	0.19
512×512	92.29%	0.69

## Data Availability

The code employed for the numerical experiments can be found at https://github.com/AleBenfe/upU-net_Perlin (accessed on 21 April 2022).
